# Book Reviews

**Published:** 1916-05

**Authors:** 


### BOOK REVIEWS

THE ENDEMIC DISEASES OF THE SOUTHERN STATES. By William H. Deaderick, M.D., and Loyd Thompson, M.D., of Hot Springs, Arkansas. Octavo volume of 546 pages with 117 illustrations. Philadelphia and London: W. B. Saunders Company, 1916. Cloth, $5.00 net; Half Morocco $6.50 net.

The Chapters of this book are Malaria, Blackwater Fever, Pellagra, Amebic Dysentery, Hook-Worm Disease and Other Intestinal Parasites. Each subject is cleverly, clearly and completely treated. Is not this statement almost an entire review of itself?

There is more to be said, however. In the first place a number of diseases having their incidence in the South are omitted because of their tropical character and the fact that they are so rapidly coming under prophylactic control that their consideration grows steadily less important. The one time sensational scourge of the South, Yellow Fever, is mentioned only to differentiate it from Hemoglobinuric Fever. Its dismissal is due to its having passed into history as a controlled malady. The silence of the book is significant and it inspires in workers the hope of making the other parasitic diseases follow it to the same limbo.

The study of Malaria is extensive and interesting. It takes up all the questions one is familiar with and gives concisely the authoritative decision of the time upon them. The literature has been sifted and to all the good things found have been added the authors' experiences. In the discussion of the diagnosis the stand is taken that while there is not the least doubt as to the role the parasite plays in the cause and development of the disease, the parasite may not always be found and the other classic signs have to be given full value. This contradicts the teaching of some enthusiastic men who have gone sc far as to say that reliance upon the therapeutic test of malaria's presence by its reaction to quinine was "criminal" and that the parasite must be found. As against this the book quotes Plehn as saying that the presence of the malarial parasites in the blood of the West African negro is of no value. The reason is that he may be suffering with something else and the malaria indication would be confusing at least. Certain it is that men in the field have to diagnose and treat malaria without the aid of the microscope in this day and generation, however much we may wish for different things, and they have their judgment supported by the considerations here found. The chapter helps them and tends to lead them to more extended study of the alluring pathways it discloses.

In Blackwater Fever the writers state that the fact it does not respond to quinine is one of the strongest evidences that it is not malaria PER SE. Here where the laboratory findings are not sufficiently clear or comprehensive to delineate a pathology, the symptom complex of the clinician is kept well to the fore and the study is proceeded with inimicable team work. The prophylaxis of the disease is brief and pointed.

As to Pellagra we do not get anywhere. The history of the disease is related; the various symptoms and destructive lesions are described; the theories of a host of men are mentioned; the disasters to the patients are deplored; the statistics of many investigators are analyzed: the word "Pellagra" is discussed, and there we are. What more could they do? Every thing of the least value that is known about the disease is given place and the very multiplicity of reference and comment shows simply that we have Pellagra and do not know what we have got. The field is still wide open and the careful searchers are many. These authors may be discoverers eventually for they have open minds and have collated for themselves and their readers all that has been done and related as to the disease.

In the diagnosis of Amebic Dysentery they say that "It is upon the microscopic examination of the feces or pus from a hepatic abscess for the ENDAMEBA HISTOLYTICA that the final diagnosis must rest."

The treatise leads up to this and the treatment depends upon it. Clinical manifestations are described and the picture made as definite as possible, but all this is to lead the student to demand for himself and his patient the early aid of the laboratory. The prognosis when the stage of abscess has been reached is bad. Prior to that time in children much can be done. The chapter will assist greatly in the prompt recognition of the infection.

Hook-Worm Disease we know almost all about. The subject is well handled and the treatment brought up to date. The laboratory findings are necessary to a diagnosis but the laboratory can be in the pocket of the man who has to go "among the sticks" to see his patient at times. The prophylaxis is easy to teach and the teacher is the family doctor.

This book should be in the hands of every man in the South as a part of his equipment in combatting disease and caring for its victims. It contains the results of the splendid work done by several organizations and by heroic men who have sacrificed themselves for the good of their fellows. It meets the time and should fill the place.

---

CANDY MEDICATION. By Bernard Fantus, M.D., Professor of Pharmacology and Therapeutics, College of Medicine, University of Illinois, Chicago. 81 pages, Cloth $1.00. St. Louis; C. V. Mosby Company.

This book is of far more importance than its title and size would indicate. It does not teach the giving of candy nor does its limit its usefulness by the brevity of its structure. It does teach and teach originally the covering of unpleasant drugs with candy so that the medicine may be easily given without needless dilution or permanent incapsulation. By "covering" we do not mean "coating." The formulae are given for the selection of various easily secured and prepared menstrua with which the drugs can be incorporated and made into tablets that will be acceptable to the child both on account of their appearance and taste. The tablets can be made inexpensively and thus the prescription is not a theory but an available matter.

The original element in this work is the teaching of real candy making in connection with the compounding of the remedies. The author has been at the pains of taking a course in candy making so as to enable him to depart from the former ideas of "confections" and to supply us with the materials that will carry the medicines without destroying or sequestrating them.

A man who dispenses his own medicines can make the tablets with a little practise. Every progressive druggist should have the apparatus and be ready to fill the orders in a brief time.

The book should be in the hands of both branches of the profession of the healing art.

